# TACE versus TACE + entecavir versus TACE + tenofovir in the treatment of HBV associated hepatocellular carcinoma

**DOI:** 10.1186/s12885-023-10694-9

**Published:** 2023-03-13

**Authors:** Haohao Lu, Chuansheng Zheng, Bin Xiong, Xiangwen Xia

**Affiliations:** 1 Department of Radiology, Union Hospital, Tongji Medical College, Huazhong University of Science and Technology, Jiefang Avenue #1277, 430022 Wuhan, China; 2 Hubei Province Key Laboratory of Molecular Imaging, 430022 Wuhan, China

**Keywords:** Nucleos(t)ide analogues, Antiviral therapy, Transcatheter arterial chemoembolization, Viral hepatitis B, Hepatocellular carcinoma, TACE

## Abstract

**Background:**

At present, there are a variety of antiviral drugs for HBV in clinical practice, but there is no standard scheme for transcatheter arterial chemoembolization(TACE) combined with antiviral drugs. The aim of this study was to investigate whether TACE must be combined with antiviral therapy in patients of HBV-related hepatocellular carcinoma(HCC). Meanwhile, the efficacy and safety of TACE combined with entecavir and TACE combined with tenofovir in the treatment of HBV-related HCC were compared.

**Method:**

This study included 536 patients with HBV-related HCC who underwent TACE in Union Hospital from March 2017 to March 2020, and they met the criteria. They were divided into three groups: control group (N = 212): TACE alone; Entecavir group (N = 220): TACE combined with entecavir; and Tenofovir group (N = 228): TACE combined with tenofovir. We conducted a retrospective study to analyze the efficacy and safety of the three groups of patients.

**Results:**

Objective response rate(ORR): 29.2% in control group, 54.1% in entecavir group, and 63.2% in tenofovir group (P < 0.05). Disease control rate(DCR): 63.7% in control group, 80.9% in entecavir group, and 88.1% in tenofovir group (P < 0.05). Median overall survival(mOS): control group, 12.2 months; entecavir group, 17.3 months; tenofovir group, 22.5 months (p < 0.05). Median progression-free survival (mPFS): control group, 9.3 months; entecavir group, 15.5 months; tenofovir group, 16.6 months (p < 0.05). At 6 months, there was an increase in creatinine(Cr) and a decrease in glomeruar filtration rate(GFR) in tenofovir group, which were statistically different from control and entecavir groups (p < 0.05).

**Conclusion:**

TACE combined with entecavir and TACE combined with tenofovir had higher ORR and DCR, longer OS and PFS than TACE alone. The OS of TACE combined with tenofovir was higher than that of TACE combined with entecavir. TACE combined with tenofovir is a safe strategy, but we cannot completely ignore the impact of tenofovir on renal function.

## Introduction

Hepatitis B virus (HBV) infection is a serious public health problem worldwide. Patients with chronic HBV infection experience fatal complications such as liver cirrhosis (LC) and hepatocellular carcinoma (HCC) [1]. HCC is one of the most common malignant tumors of digestive system, which threatens people’s life and health. At present, HCC is the fourth most common malignant tumor and the second cause of tumor death in China. There are many causes of HCC. HBV infection is one of the most important causes of HCC in China [2], and approximately 75% of HCC patients are infected with HBV. HBV is one of the important factors in the occurrence, development and prognosis of HCC [3]. HBV leads to the occurrence of HCC through different mechanisms. It causes disease through the three-step pathogenesis of hepatitis-cirrhosis-HCC. It can also directly embed host genes to lead to cell carcinogenesis, transactivate a variety of oncogenes through HBx [4], inhibit tumor suppressor genes and directly stimulate cell growth, inhibit DNA repair in damaged cells and other mechanisms. As the most important risk factor of HCC, HBV infection often synergizes with other risk factors to promote the occurrence of cirrhosis, which leads to the formation of HCC. Because HCC has no characteristic symptoms in the early stage, most patients are already in the advanced stage when they are diagnosed with HCC. Although surgical resection is the first treatment option for patients with HCC, most patients have lost the opportunity for surgery when HCC is found. Studies have reported that only 20–25% of the patients with HCC have the chance of surgery. Transcatheter arterial chemoembolization (TACE) was first proposed by Yamada in 1978. For advanced HCC, TACE is one of the effective treatments [5]. The main principle of TACE is the transcatheter injection of chemotherapeutic agents and embolic agents into the tumor tissue. Although the efficacy of TACE is continuously improving with the continuous update of interventional instruments, embolization materials and operation techniques [6], the side effects of TACE should not be ignored. Many studies have reported that TACE damages normal liver tissue and inhibits the immune function of the body while killing tumors, which may lead to the reactivation of HBV [7], aggravate liver injury, and even lead to liver failure. Studies have reported that the level of HBV-DNA is an independent predictor of HCC recurrence [8] and one of the factors affecting the long-term outcome of TACE [9]. At present, nucleos (t) ide analogues are the main anti-HBV drugs, which competitively inhibit the entry of nucleotides into the viral DNA strand and interfere with the transcription and synthesis of the virus by inhibiting the activity of viral DNA synthase. Mechanistically, TACE combined with antiviral therapy can delay HBV-induced cirrhosis progression and also prevent viral reactivation after TACE. The concept of TACE combined with antiviral therapy has been accepted by more and more scholars. However, with the development of antiviral drugs for nucleos(t)ide analogues, many new antiviral drugs have emerged. Currently, there is no standard treatment regimen for TACE combined with antiviral therapy [10]. The most commonly used antiviral drugs in clinic are entecavir (ETV) and tenofovir (TDF). The aim of this study was to investigate whether TACE must be combined with antiviral therapy in patients of HBV-related hepatocellular carcinoma(HCC). Meanwhile, the efficacy and safety of TACE combined with entecavir and TACE combined with tenofovir in the treatment of HBV-related HCC were compared.

## Materials and methods

### General information

The data of 536 patients with HBV-related hepatocellular carcinoma who underwent TACE in the department of intervention, Union Hospital, Tongji Medical College, Huazhong University of Science and Technology from March 2017 to March 2020 were collected. Inclusion criteria (1) Meet the diagnostic criteria of HCC, including clinical diagnosis or pathological diagnosis; (2) Hepatitis B virus infection, regardless of their stage of HBV infection; (3) Patients aged 18–70 years; (4) Meet the indications of TACE treatment, liver function classification Child-Pugh A-B, physical score (ECOG) 0–2 points; (5) Survival time expected ≥ 3 months; (6) Complete clinical follow-up data. Exclusion criteria: (1) There are contraindications to TACE; (2) Liver function classification Child-Pugh C, performance score (ECOG) > 2 points; (3) Patients with other hepatitis; (4) With other vital organ dysfunction, such as heart, kidney dysfunction; (5) Recently received tumor-related treatment and antiviral therapy. Patients were divided into three groups according to their antiviral therapy: control group (N = 212): TACE alone without antiviral therapy; entecavir group (N = 220): TACE combined with entecavir; and tenofovir group (N = 228): TACE combined with tenofovir. The baseline data of patients were collected, including gender, age, alanine aminotransferase (ALT), aspartate aminotransferase (AST), total bilirubin, albumin, HBV-DNA, preoperative Child-Pugh classification of liver function, BCLC stage, ECOG score, urea nitrogen (BUN), creatinine (Cr) and glomerular filtration rate (GFR).

Clinical diagnostic criteria for HBV related HCC [11]: if the diameter of intrahepatic lesions is > 2 cm, at least one imaging examination has typical characteristics of hepatocellular carcinoma; If the diameter of intrahepatic lesions is ≤ 2 cm, at least two imaging examinations have typical features of hepatocellular carcinoma.

### Method

#### TACE process

The patient lies supine position with the inguinal region disinfected and draped aseptically. Local anesthesia was performed at the puncture site using 2% lidocaine, the femoral artery was punctured using the Seldinger technique, and a 5 F catheter sheath was placed. A 5 F Yashiro catheter was inserted into the celiac trunk and superior mesenteric artery for arteriography to identify the feeding artery of the tumor. A 2.7 F microcatheter was then superselectively cannulated into the feeding artery of HCC. After reaching the target vessel, an emulsion of lipiodol + epirubicin was injected first, followed by an appropriate amount of gelatin sponge particles. At the end of the treatment, the catheter was removed and the puncture site was pressurized and dressed. The patient received enhanced CT or MRI every 6–8 weeks, and re-TACE was determined according to re-examination conditions.

#### Antiviral therapy

The control group did not receive antiviral therapy. In the entecavir group, oral entecavir, 0.5 mg, QD, was started at one week before TACE. In the Tenofovir group, oral tenofovir, 300 mg, QD, was started at one week before TACE. Antiviral drugs were taken for a long time.

### Outcome measures

Primary study endpoints: (1) All patients in the three groups were evaluated for tumor response after 3 months of treatment using mRECIST criteria [12], including complete response (CR), partial response (PR), stable disease (SD), and progressive disease (PD); (2) Objective response rate (ORR) and disease control rate (DCR) of tumors in the three groups; (3) Overall survival (OS) and progression-free survival (PFS) of patients in the three groups.

Secondary study endpoints: (1) Changes of liver function, renal function and HBV-DNA in the three groups after treatment; (2) Comparison of HBV-DNA negative rate and reactivation rate after treatment among the three groups.

Definition of HBV conversion: DNA level decreased below the lower limit of detection (< 100 IU/ml).

Definition of HBV reactivation: Increase in DNA level to more than 10-fold of baseline level.

### Statistical methods

Statistical analysis was performed by using SPSS 24.0 software. Number of cases (percentage) was used for enumeration data, and chi-square test (Pearson Chi-Square) was used for difference. Measurement data were expressed as mean ± standard deviation, and one-way ANOVA test was used. OS and PFS were shown by Kaplan-Meier curves, and the Log-Rank test was used for the comparison of OS and PFS among the three groups. P < 0.05 was considered to indicate a statistically significant difference.

## Results

(1) Comparison of pretreatment enumeration data among the three groups (Table 1).

**Table 1:** Comparison of baseline data among three groups

			Group			p-value	
			Control group (N = 212)	ETV group (N = 220)	TDF group (N = 228)	Chi-Square Tests	Oneway ANOVA
Gender	Female	Count(%)	67(31.6%)	64(29.1%)	75(32.9%)	0.880	
	Male	Count(%)	145(68.4%)	156(70.9%)	153(67.1%)		
Child-Pugh classification of liver function	A	Count(%)	140(66.0%)	139(63.2%)	135(59.2%)	0.781	
	B	Count(%)	72(34.0%)	81(36.8%)	93(40.8%)		
BCLC staging	A	Count(%)	25(11.8%)	27(12.3%)	20(8.8%)	0.896	
	B	Count(%)	120(56.6%)	112(50.9%)	133(58.3%)		
	C	Count(%)	67(31.6%)	81(36.8%)	75(32.9%)		
ECOG score	0	Count(%)	148(69.8%)	145(65.9%)	154(67.5%)	0.755	
	1	Count(%)	41(19.4%)	47(21.4%)	43(18.9%)		
	2	Count(%)	23(10.8%)	28(12.7%)	31(13.6%)		
Levels of HBV-DNA (IU/ml)	> 2000	Count(%)	100(47.2%)	133(60.5%)	123(53.9%)	0.081	
	100–2000	Count(%)	74(34.9%)	61(27.7%)	74(32.5%)		
	< 100	Count(%)	38(17.9%)	26(11.8%)	31(13.6%)		
Age(Years)	Mean ± SD		47.8 ± 12.1	51.3 ± 15.4	56.1 ± 18.7		0.433
ALT before treatment(U/L)	Mean ± SD		51.4 ± 26.3	55.6 ± 21.5	59.2 ± 20.7		0.210
AST before treatment(U/L)	Mean ± SD		62.3 ± 20.6	57.6 ± 17.1	61.5 ± 22.4		0.327
Bilirubin before treatment(µmol/L)	Mean ± SD		19.6 ± 11.3	21.4 ± 13.9	20.7 ± 8.5		0.155
HBV-DNA(lgX IU/ml)	Mean ± SD		4.86 ± 1.75	4.53 ± 2.36	4.69 ± 2.08		0.408
Albumin(g/L)	Mean ± SD		32.62 ± 4.60	33.18 ± 3.91	32.15 ± 4.27		0.371
BUN(mmol/L)	Mean ± SD		5.79 ± 2.21	6.12 ± 1.93	5.96 ± 2.16		0.256
Cr(µmol/L)	Mean ± SD		82.11 ± 25.34	79.56 ± 23.71	75.44 ± 27.23		0.568
GFR(ml/min)	Mean ± SD		98.76 ± 19.75	104.49 ± 21.02	101.52 ± 25.63		0.309

Shown in Table 1, there was no significant difference in gender, liver function grade, levels of HBV-DNA, BCLC stage and ECOG score among the three groups using chi-square test (P > 0.05).

(2) Comparison of measurement data before treatment among the three groups(Table 1).

The mean ± standard deviation of age, ALT, AST, total bilirubin, albumin, HBV-DNA, BUN, Cr, and GFR in the three groups are shown in Table 1. One-way ANOVA test was used for comparison among three groups, with P > 0.05, without statistical difference.

(3) Comparison of liver function, renal function, and HBV-DNA among the three groups at 2 months of treatment (Table 2).

**Table 2:** Comparison of liver and kidney function and HBV-DNA among the three groups at 2 months, 4 months and 6 months after treatment

Comparison of blood parameters after treatment							
		2 months		4 months		6 months	
		Mean ± SD	Oneway ANOVA	Mean ± SD	Oneway ANOVA	Mean ± SD	Oneway ANOVA
ALT(U/L)	Control group	73.3 ± 33.2	0.167	87.5 ± 29.4	0.041	75.1 ± 31.5	0.039
	ETV group	56.2 ± 37.6		62.7 ± 35.1		51.5 ± 29.7	
	TDF group	58.5 ± 30.4		61.3 ± 27.3		46.8 ± 22.6	
AST(U/L)	Control group	72.0 ± 31.1	0.103	76.4 ± 28.5	0.032	74.3 ± 24.7	0.028
	ETV group	57.8 ± 25.5		52.6 ± 22.0		47.1 ± 21.9	
	TDF group	55.2 ± 23.8		44.3 ± 19.6		45.4 ± 18.5	
Bilirubin(µmol/L)	Control group	30.6 ± 13.4	0.041	34.1 ± 35.7	0.036	42.8 ± 33.2	0.014
	ETV group	22.3 ± 12.6		21.7 ± 19.2		24.2 ± 17.3	
	TDF group	21.7 ± 15.3		22.0 ± 11.5		21.4 ± 15.8	
BUN(mmol/L)	Control group	5.59 ± 2.84	0.651	5.63 ± 2.06	0.498	5.74 ± 2.18	0.534
	ETV group	5.81 ± 1.99		5.78 ± 2.03		5.92 ± 2.31	
	TDF group	5.93 ± 1.78		5.80 ± 2.25		6.01 ± 3.11	
Cr(µmol/L)	Control group	79.26 ± 19.78	0.612	80.45 ± 17.57	0.325	78.63 ± 21.02	0.040
	ETV group	78.18 ± 20.15		76.59 ± 19.16		79.35 ± 18.84	
	TDF group	76.74 ± 23.42		79.82 ± 24.39		95.93 ± 26.37	
GFR(ml/min)	Control group	101.37 ± 26.25	0.412	103.04 ± 22.76	0.215	101.79 ± 17.33	0.031
	ETV group	100.62 ± 32.18		101.45 ± 28.21		105.13 ± 21.42	
	TDF group	99.83 ± 35.40		94.26 ± 39.54		82.65 ± 27.31	
HBV-DNA(lgX IU/ml)	Control group	4.95 ± 2.06	0.012	5.28 ± 1.91	0.008	5.33 ± 2.25	0.000
	ETV group	3.16 ± 1.42		2.24 ± 1.36		1.30 ± 0.94	
	TDF group	2.89 ± 1.75		2.07 ± 1.57		1.15 ± 0.79	

There was no statistically significant difference in ALT, AST, BUN, Cr, and GFR among the three groups, with P > 0.05. There were statistically significant differences in bilirubin and HBV-DNA among the three groups of patients treated for 2 months (one-way ANOVA test, **P < 0.05**).

(4) Comparison of liver function, renal function and HBV-DNA among the three groups at 4 months of treatment (Table 2).

There was no significant difference in BUN, Cr and GFR among the three groups (P > 0.05). There were statistically significant differences in ALT, AST, bilirubin and HBV-DNA among the three groups of patients treated for 4 months (one-way ANOVA test, **P < 0.05**).

(5) Comparison of liver function, renal function, HBV-DNA among the three groups at 6 months of treatment (Table 2).

There was no significant difference in BUN among the three groups, P > 0.05. There were statistically significant differences in ALT, AST, bilirubin, Cr, GFR and HBV-DNA among the three groups of patients treated for 6 months (one-way ANOVA test, **P < 0.05**).

(6) Tumor response evaluation after 3 months of treatment in the three groups (Table 3).

**Table 3:** Efficacy evaluation of tumor response in the three groups at 3 months of treatment

			Group			Chi-square test (p-value)
			Control group	ETV group	TDF group	Pearson Chi-Square
Response evaluation	CR	Count(%)	10(4.7%)	19(8.6%)	25(11.0%)	0.010
	PR	Count(%)	52(24.5%)	100(45.5%)	119(52.1%)	
	SD	Count(%)	73(34.5%)	59(26.8%)	57(25.0%)	
	PD	Count(%)	77(36.3%)	42(19.1%)	27(11.9%)	
	ORR	Count(%)	62(29.2%)	119(54.1%)	144(63.2%)	0.001
	DCR	Count(%)	135(63.7%)	178(80.9%)	201(88.1%)	0.002

Control group: 10 patients (4.7%) with CR, 52 patients (24.5%) with PR, 73 patients (34.5%) with SD and 77 patients (36.3%) with PD. Entecavir group: 19 (8.6%) patients with CR, 100 (45.5%) patients with PR, 59 (26.8%) patients with SD and 42 (19.1%) patients with PD. Tenofovir group: 25 (11.0%) patients achieved CR, 119 (52.1%) patients achieved PR, 57 (25.0%) patients achieved SD, and 27 (11.9%) patients achieved PD.

ORR refers to the rate of patients achieving CR and PR. DCR refers to the rate of patients achieving CR, PR, and SD. The ORR was 29.2% in the control group, 54.1% in the entecavir group, and 63.2% in the tenofovir group. The DCR was 63.7% in the control group, 80.9% in the entecavir group, and 88.1% in the tenofovir group.

As shown in Table 3, the comparison between the three groups using the chi-square test, **P < 0.05**, the result of entecavir group and tenofovir group were better than that of the control group, with a statistically significant difference.

(7) The conversion rate and reactivation rate of HBV-DNA in the three groups (Table 4).

**Table 4:** HBV-DNA conversion rate and reactivation rate at 2 months, 4 months, and 6 months in the three groups

		Group			Chi-square test (p-value)
		Control group	ETV group	TDF group	Pearson Chi-Square
DNA conversion rate	2 months	2.2%	23.7%	31.3%	0.003
	4 months	3.5%	41.2%	45.8%	
	6 months	6.4%	62.9%	71.2%	
DNA reactivation rate	2 months	22.6%	8.7%	7.9%	0.001
	4 months	56.3%	10.1%	8.2%	
	6 months	61.1%	11.3%	10.4%	

DNA conversion rate: at 2 months, 2.2% in the control group, 23.7% in the entecavir group, and 31.3% in the tenofovir group; at 4 months, 3.5% in the control group, 41.2% in the entecavir group, and 45.8% in the tenofovir group; at 6 months, 6.4% in the control group, 62.9% in the entecavir group, and 71.2% in the tenofovir group. As shown in Table 4, Chi-square test was used for comparison between the three groups, **P < 0.05**, and the DNA conversion rate in the entecavir and tenofovir groups was significantly higher than that in the control group, with a statistically significant difference.

DNA reactivation rate: at 2 months, 22.6% in the control group, 8.7% in the entecavir group, and 7.9% in the tenofovir group; at 4 months, 56.3% in the control group, 10.1% in the entecavir group, and 8.2% in the tenofovir group; at 6 months, 61.1% in the control group, 11.3% in the entecavir group, and 10.4% in the tenofovir group. As shown in Table 4, Chi-square test was used for comparison among the three groups, with **P < 0.05**. The DNA reactivation rate in entecavir group and tenofovir group was significantly lower than that in control group, with statistical difference.

**Fig. 1:**
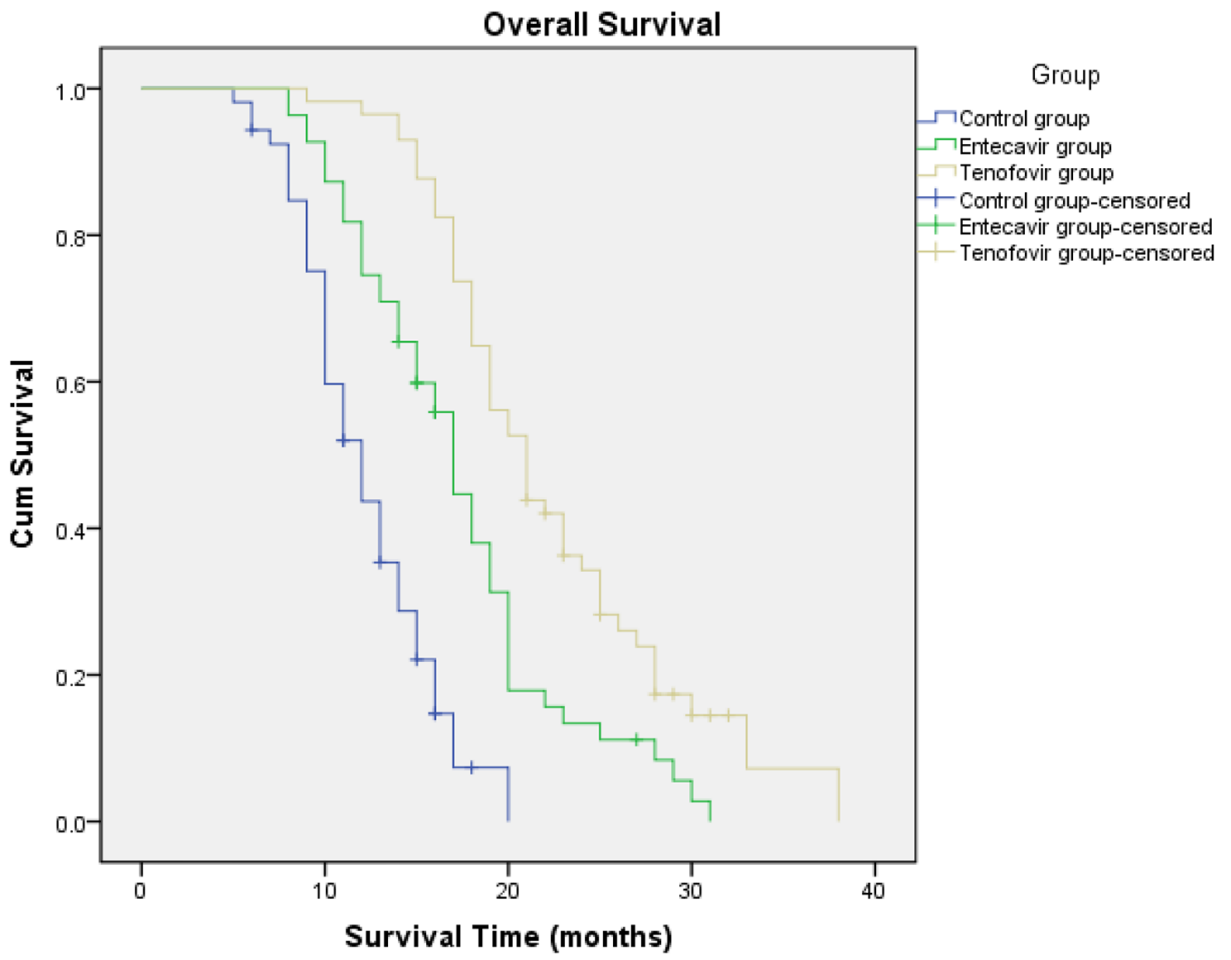
Overall survival of patients in three groups mOS: Control group,12.2 months (95% CI 11.2–13.3 months); Entecavir group,17.3 months (95% CI 15.6–18.9 months); Tenofovir group,22.5 months (95% CI 20.5–24.4 months)

**Fig. 2:**
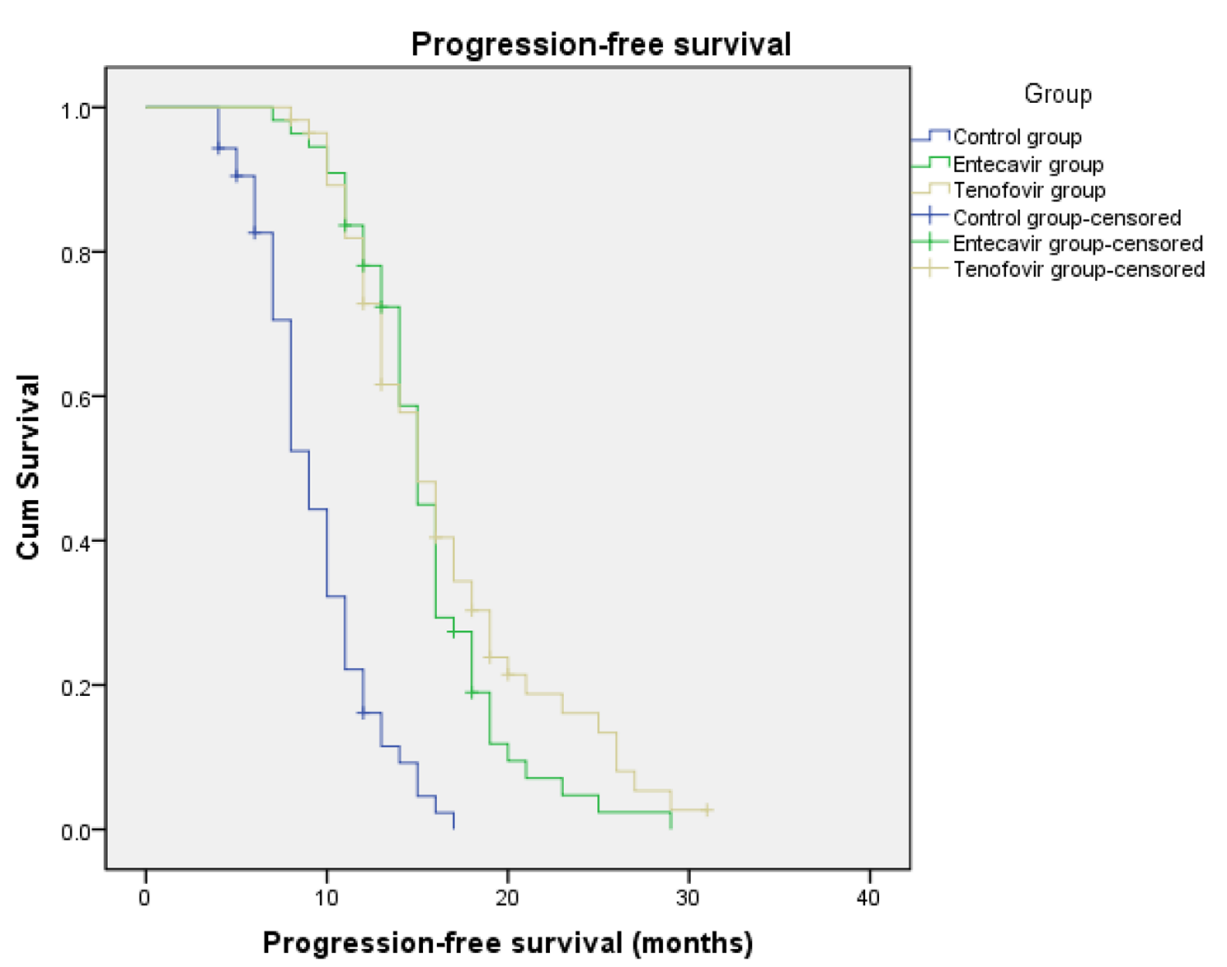
Progression-free survival time in the three groups mPFS: Control group,9.3 months (95% CI 8.4–10.2 months); Entecavir group,15.5 months (95% CI 14.3–16.6 months); Tenofovir group,16.6 months (95% CI 15.1–18.2 months)

(8) OS and PFS in the three groups (Figs. 1 and 2).

mOS: control group, 12.2 months (95% CI 11.2 to 13.3 months); entecavir group, 17.3 months (95% CI 15.6 to 18.9 months); tenofovir group, 22.5 months (95% CI 20.5 to 24.4 months). As shown in Fig. 1, Log-Rank test was used between the three groups. The tenofovir group was superior to entecavir group (**P < 0.05**) and entecavir group was superior to control group (**P < 0.05**).

mPFS: control group, 9.3 months (95% CI 8.4–10.2 months); entecavir group, 15.5 months (95% CI 14.3–16.6 months); tenofovir group, 16.6 months (95% CI 15.1–18.2 months). As shown in Fig. 2, Log-Rank test was used among the three groups. The entecavir and tenofovir groups were superior to the control group (**P < 0.05**), and there was no statistically significant difference between the entecavir and tenofovir groups (P > 0.05).

## Discussion

The pathogenesis of HCC is not fully understood, and its pathophysiological process is a complex process in which a variety of pathogenic factors act together. At present, it is believed that carcinogens such as viral hepatitis, cirrhosis, environmental factors, living habits and aflatoxin are closely related to the occurrence of HCC. In different regions of the world, the main pathogenic factors of HCC vary [13–15].In China, the most important risk factor for the development of HCC is HBV infection. Studies have reported that HBV virus carriers account for about 10% in the Chinese population, and about 75% of hepatocellular carcinomas occur in association with HBV. About 0.4–0.6% of chronic HBV(CHB) infections progress and are definitely diagnosed with HCC, and the probability of HBV infection to liver cancer is 30–35 times that of non-HBV infection. Therefore, active antiviral therapy can delay the development of cirrhosis and reduce the incidence of liver cancer. The study found that the cumulative incidence of HCC in CHB patients with serum HBV DNA ≥ 2000 IU/ml was significantly higher than that in CHB patients with serum HBV DNA < 2000 IU/ml [16]. Won-Mook Choi et al. reported [17] that for patients with high viral load (≥ 8.00 lg IU/mL), early initiation of antiviral therapy can maintain the lowest risk of HCC development in these patients. Several studies have reported [18–20] that antiviral therapy in patients with chronic hepatitis B significantly reduces the incidence of HCC.

There are also many comparative studies on whether the risk of HCC varies after the use of different antiviral drugs. Some scholars have reported [21, 22] that tenofovir can significantly reduce the incidence of HCC compared with entecavir in patients with chronic hepatitis B. However, some researchers have different conclusions, and their study reported [23, 24] that both tenofovir and entecavir can reduce the occurrence of HCC in HBV patients and prolong their survival, with no statistically significant difference. Bao-Hong Yuan et al. reported [25] that in Asian populations, tenofovir was recommended, which significantly reduced the occurrence of HCC compared with entecavir.

HBV infection leads to the further development of HCC, destroys hepatocytes, and weakens the killing effect on cancer cells. By destroying the pericellular matrix and continuously neovascularizing, hepatocellular carcinoma cells acquire the ability of invasion and metastasis, which is the mechanism of HCC progression to the middle and advanced stages. Studies have reported that HBV-DNA load is negatively correlated with the survival of HCC, and the HBV-DNA load is associated with a worse prognosis [26]. Whether antiviral therapy is required for patients with HBV-related HCC, recent studies have agreed that antiviral therapy is necessary for patients with HBV-related HCC. Among the many nucleos(t)ide analogues of antiviral drugs, entecavir and tenofovir are the most commonly used first-line recommended anti-HBV drugs, which can effectively delay the development of cirrhosis and reduce the incidence of HCC. Lingling He et al. found [27] that for patients with unresectable HBV-related HCC, receiving antiviral therapy, improved OS and PFS of patients. Junyi Shen et al. reported [28] that HCC patients with ultra-Milan criteria had a high recurrence rate after hepatectomy, and tenofovir could significantly reduce the recurrence rate after hepatectomy compared with entecavir. Ji Hyun Lee et al. reported [29] that the use of both entecavir and tenofovir reduced the recurrence rate (aHR = 1.038, P = 0.963) and mortality (aHR = 0.799, P = 0.431) in patients with HBV-related HCC after radical treatment, with no statistically significant difference.

This study showed that HBV-DNA turned negative in 62.9% of the entecavir group and 71.2% of the tenofovir group at 6 months of treatment, which could effectively reduce the viral load and was consistent with the results reported in other studies [30]. Nucleos(t)ide analogues protect residual liver cells from hepatitis B virus destruction by inhibiting the process of viral replication, reducing the amount of hepatitis virus, and compensating normal liver tissue as much as possible, which is conducive to the recovery of liver function in patients. The level of liver function is a key factor that directly affects the survival and prognosis of patients with HCC. In the whole treatment process of HCC, it is also necessary to protect the liver function of patients, so that patients are likely to receive active and effective comprehensive treatment of HCC. It is reported by Su-Su Zhang [31] that TACE significantly increased the risk of HBV reactivation (OR: 3.70; 95% CI:1.45–9.42; P < 0.01), and prophylactic antiviral therapy had a protective effect. Enze Jiang et al. reported [32] that antiviral therapy can reduce viral toxicity, prevent viral reactivation, reduce liver inflammation, reverse cirrhosis, reduce the risk of recurrence and metastasis of HCC, and prolong the survival of patients, and antiviral therapy should be used as a conventional combination therapy for patients with HBV-related HCC.

In this study, ALT, AST, and bilirubin levels were significantly better in the entecavir and tenofovir groups at both 4- and 6-month follow-up. For advanced HCC, TACE is one of the commonly used treatments. Injection of chemotherapeutic drugs and embolic agents through the catheter have killing and inhibitory effects on HCC, but they also affect the blood supply of the liver and reduces immune function. This will destroy the normal liver tissue and reduce the inhibitory effect on the virus. This may lead to massive replication of hepatitis B virus after TACE, causing acute liver injury or other complications, and even leading to liver failure and life-threatening. Sun Hong Yoo [33] found that TACE combined with active antiviral therapy could reduce the risk of liver function deterioration and enable patients to continue anti-tumor treatment, thereby improving the survival of patients. Kai Wang et al. reported [34] that even HCC patients with negative HBV-DNA are still associated with the risk of HBV reactivation after TACE, while antiviral therapy can reduce the risk of reactivation and help improve liver function after TACE. The results of this study showed that the virus conversion rate at 6 months in the control group was 6.4%, which was significantly lower than that in the entecavir and tenofovir groups. The reactivation rate of HBV-DNA in the control group was 22.6% at 2 months, 56.3% at 4 months, and 61.1% at 6 months, which was significantly higher than that in the entecavir and tenofovir groups; the analysis may be caused by decreased liver blood supply and decreased immune function after TACE. The massive replication of the virus leads to liver function damage in patients, so the results of this study showed that the liver function parameters at 4 and 6 months in the control group were statistically significantly worse than those in the entecavir and tenofovir groups. Chia-Chi Wang reported [35] that the risk of HBV reactivation after TACE is high, so HCC patients with high viral load (HBV DNA > 2000IU/mL) are recommended to receive antiviral therapy after radical treatment or TACE. Xing Li et al. [36] reported that in patients with HBV-related HCC undergoing TACE, virological events (6.8%vs54.4%, p = 0.000) and HBV viral reactivation (0.0%vs11.6%, p = 0.039) were significantly reduced in the group receiving entecavir therapy compared with the control group. Xiang-Ming Lao et al. reported [37] that among 172 patients with HBV-related HCC receiving TACE, 33 patients (14.5%) had postoperative HBV activation.

Many studies have reported that whether patients receive antiviral therapy is one of the factors affecting the medium- and long-term efficacy of TACE. Therefore, TACE combined with antiviral therapy is now a respected treatment regimen in clinical practice and the direction of many scholars’ research. Qi-Feng Chen et al. reported [38] that TACE should be combined with systemic therapy, such as moleculartargeted therapy, immunotherapy and antiviral therapy. TACE combined with antiviral therapy can significantly improve the survival of HBV-related HCC patients [39–41]. Myung Pyo Kim et al. reported [42] that for patients with low viral load (DNA level < 2000 IU/ml) receiving TACE, antiviral therapy was a prognostic factor for two-year survival (HR,0.503,P = 0.022). It is reported by Weili Qi [43] that the combination of tenofovir significantly reduced the recurrence rate (HR,0.64;95%CI,0.49–0.83; p = 0.001) and mortality (HR,0.32;95%CI,0.20–0.50;p < 0.001) compared with entecavir in patients with HBV-related HCC after surgical resection.

The results of this study showed that the ORR and DCR in both the entecavir group and the tenofovir group were better than those in the control group (P < 0.05), which was also consistent with the results of other studies. Su Jong Yu et al. found [44] that patients with high levels of HBV-DNA before TACE had poor overall survival rate and faster tumor progression, and the cause of death was not hepatitis outbreak but tumor progression. However, there is no uniform treatment regimen for TACE combined with antiviral therapy. The Wenbo Shao study reported [45] that even patients with low viral load are at risk of HBV activation after TACE, so antiviral therapy is very important. There are many anti-HBV therapeutic drugs on the market, combined with different antiviral drugs, which may affect the efficacy of patients. The results of this study showed that the entecavir and tenofovir groups were superior to the control group in terms of PFS (P < 0.05). In the control group, mPFS was 9.3 months; in the entecavir group, mPFS was 15.5 months; and in the tenofovir group, mPFS was 16.6 months; however, there was no statistical difference between the entecavir and tenofovir groups. In terms of OS, there was a statistically significant difference among the three groups (P < 0.05). In the control group, the mOS was 12.2 months; in the entecavir group, the mOS was 17.3 months; and in the tenofovir group, the mOS was 22.5 months; the tenofovir group was superior to the entecavir group (P < 0.05) and the entecavir group was superior to the control group (P < 0.05). The reason may be that tenofovir has stronger viral inhibition ability and DNA conversion rate, and also has the effect of regulating the local immune environment of the liver. ETV is a nucleoside analogue that inhibits viral replication mainly by competing with HBV for the site of DNA polymerase action, which can rapidly inhibit the replication of HBV-DNA and promote ALT normalization in a short period of time, but the immunoregulatory effect of nucleoside analogues is weak and the number of IFN-λ3 induced in the body is small. TDF is a nucleotide analogue that can rapidly inhibit viral replication, effectively reduce the level of serum HBsAg, and induce an increase in serum IFN-λ3 levels, which has an immunoregulatory effect. Several studies have reported [46–48] that patients with HBV-related HCC have longer OS and lower recurrence rates with the combination of tenofovir compared with entecavir after radical treatment. Sung Won Lee [49] found that TDF directly ameliorates liver fibrosis by downregulating the PI3K/Akt/mTOR signaling pathway, which leads to apoptosis of activated HSC. The anti-fibrotic effect of TDF, which can delay the development of cirrhosis, may also be the reason for longer OS after TACE in the TDF group.

Although studies have shown that compared with entecavir, tenofovir can quickly inhibit virus replication and reduce the level of serum HBsAg. However, some research results have confirmed that the plasma stability of TDF is poor. After entering the human body, TDF is quickly hydrolyzed into TFV (tenofovir, TFV) with low bioavailability. A large amount of TFV accumulates around the proximal tubule, which can cause the damage of proximal tubule mitochondria, thus causing the metabolic disorder of tubular cells, and the decline of renal tubular reabsorption and secretory function. Therefore, CHB patients treated with TDF for a long time will have varying degrees of renal function damage. Young Eun Chon report [50]that long-term use of TDF may result in slow decline of renal function compared with ETV. Therefore, if the patient needs long-term treatment, the patient’s renal function must be monitored. The results of this study showed that after 6 months of treatment, the level of Cr in TDF group was higher and the GFR was lower than that in control group and ETV group. According to Chan young Jung’study [51], the risk of renal function decline of TDF is higher than that of ETV during the treatment of patients with CHB. In this study, there were significant changes in renal function indexes in TDF group. The contrast agent and chemotherapy drugs used in TACE treatment will affect the renal function of patients. During the follow-up of patients, the enhanced CT examination may also affect the renal function of patients every 6–8 weeks. In TDF group, patients received TACE 1–3 times and CT 3–4 times within 6 months. This is similar to control group and ETV group, with no significant difference. Therefore, the main possible reason for the change of renal function in TDF group is the use of tenofovir. Although changes in renal function parameters occurred in TDF group, they were slight and did not affect the continuation of treatment.

## Conclusion

For advanced hepatocellular carcinoma associated with hepatitis B, TACE combined with antiviral therapy is a recommended treatment regimen in patients without contraindications to TACE. TACE combined with entecavir and TACE combined with tenofovir had higher ORR and DCR, longer OS and PFS than that of TACE alone. TACE combined with tenofovir has longer OS than TACE combined with entecavir, which is a recommended combination. Tenofovir has a slight impact on renal function and does not affect the continuation of treatment. TACE combined with tenofovir is a safe strategy, but we cannot completely ignore the impact of tenofovir on renal function. We suggest that the renal function of patients should be monitored regularly during TACE combined with tenofovir treatment if necessary.

The shortcoming of this study is that the data are from a single center. As a result, it is a retrospective study with limited sample size. It is feasible for the prospective study with multicenter and large-sample at a later stage, which will provide more help for clinical work.

## Data Availability

The datasets used and analysed during the current study are available from the corresponding author on reasonable request.
